# Distinct but Spatially Overlapping Intestinal Niches for Vancomycin-Resistant *Enterococcus faecium* and Carbapenem-Resistant *Klebsiella pneumoniae*

**DOI:** 10.1371/journal.ppat.1005132

**Published:** 2015-09-03

**Authors:** Silvia Caballero, Rebecca Carter, Xu Ke, Bože Sušac, Ingrid M. Leiner, Grace J. Kim, Liza Miller, Lilan Ling, Katia Manova, Eric G. Pamer

**Affiliations:** 1 Immunology Program and Infectious Disease Service, Memorial Sloan-Kettering Cancer Center, New York, New York, United States of America; 2 Immunology and Microbial Pathogenesis Program, Weill Cornell Graduate School of Medical Sciences, New York, New York, United States of America; 3 Molecular Cytology Core Facility, Sloan-Kettering Institute, New York, New York, United States of America; 4 Lucille Castori Center for Microbes, Inflammation and Cancer, Memorial Sloan-Kettering Cancer Center, New York, New York, United States of America; University of California, Davis, UNITED STATES

## Abstract

Antibiotic resistance among enterococci and γ-proteobacteria is an increasing problem in healthcare settings. Dense colonization of the gut by antibiotic-resistant bacteria facilitates their spread between patients and also leads to bloodstream and other systemic infections. Antibiotic-mediated destruction of the intestinal microbiota and consequent loss of colonization resistance are critical factors leading to persistence and spread of antibiotic-resistant bacteria. The mechanisms underlying microbiota-mediated colonization resistance remain incompletely defined and are likely distinct for different antibiotic-resistant bacterial species. It is unclear whether enterococci or γ-proteobacteria, upon expanding to high density in the gut, confer colonization resistance against competing bacterial species. Herein, we demonstrate that dense intestinal colonization with vancomycin-resistant *Enterococcus faecium* (VRE) does not reduce *in vivo* growth of carbapenem-resistant *Klebsiella pneumoniae*. Reciprocally, *K*. *pneumoniae* does not impair intestinal colonization by VRE. In contrast, transplantation of a diverse fecal microbiota eliminates both VRE and *K*. *pneumoniae* from the gut. Fluorescence *in situ* hybridization demonstrates that VRE and *K*. *pneumoniae* localize to the same regions in the colon but differ with respect to stimulation and invasion of the colonic mucus layer. While VRE and *K*. *pneumoniae* occupy the same three-dimensional space within the gut lumen, their independent growth and persistence in the gut suggests that they reside in distinct niches that satisfy their specific *in vivo* metabolic needs.

## Author Summary

Intestinal colonization precedes the development of disseminated infections and bacteremia by the nosocomial pathogens vancomycin-resistant *Enterococcus* (VRE) and carbapenem-resistant *Klebsiella pneumoniae*.

Although antibiotic treatment renders mice susceptible to dense colonization by VRE or *K*. *pneumoniae*, it is unclear whether these microbes compete for space and resources in the gut. Our quantitative studies demonstrate that the density of intestinal colonization by either VRE or *K*. *pneumoniae* is unaffected by the presence of the other species, suggesting that they occupy separate niches. Using fluorescence *in situ* hybridization, we show that both bacterial species indeed occupy distinct niches but inhabit the same regions within the intestine. We find that *K*. *pneumoniae*, but not VRE, induces mucus production and invades the mucus layer adjacent to colonic epithelial cells, potentially leading to increased *K*. *pneumoniae* translocation to mesenteric lymph nodes. Despite their high colonization levels, both VRE and *K*. *pneumoniae* can be displaced from the intestinal lumen following transplantation of a healthy microbiota. Our study provides insight into the interactions between VRE and *K*. *pneumoniae* with each other and with their host.

## Introduction

Antibiotic-resistant bacteria such as vancomycin-resistant *Enterococcus faecium* (VRE) and multi-drug resistant *Klebsiella pneumoniae* represent a growing concern in hospitals worldwide. In the United States, *Enterococcus* spp. and *K*. *pneumoniae* account for nearly 10% of all hospital-acquired infections and are common causes of bacteremia [1]. Vancomycin resistance among enterococci has markedly increased since it was first described in the mid-1980s [2]. Even more alarming, however, is the increasing prevalence of carbapenem-resistant *Enterobacteriaceae*, primarily *K*. *pneumoniae*, rendering treatment of these infections very challenging [3]. Broad-spectrum antibiotic exposure, immune suppression and intravascular devices increase the risk for colonization and infection with one or more antibiotic-resistant bacteria [4]. In hospitalized patients, the intestine can become densely colonized with drug-resistant organisms. While colonization by itself does not directly cause disease, in the event of injury to the mucosal barrier colonizing bacteria may translocate beyond the intestinal tract, leading to deep tissue and bloodstream infections [5]. Consistent with this, studies have shown that intestinal colonization precedes bloodstream infection with VRE or *K*. *pneumoniae*, suggesting that loss of colonization resistance represents an early step in the progression of these infections [6–8].

Colonization resistance refers to the ability of the microbiota to prevent expansion and persistence of exogenously acquired bacterial species, a pivotal defense mechanism that can be impaired by antibiotic treatment [9,10]. Changes in microbiota composition, mainly the elimination of specific groups of anaerobic bacteria, lead to VRE domination of the gastrointestinal tract in antibiotic-treated mice [7, 11]. Similarly, colonization of mice with *K*. *pneumoniae* is facilitated by antibiotic administration [12, 13]. Antibiotic-mediated depletion of commensals also decreases production of mucus and antimicrobial effector molecules, potentially increasing the risk for bacterial invasion of the intestinal epithelium [14–18].

The interactions between different bacterial species in the gut are complex. Studies of mice singly colonized with *Bacteroides thetaiotaomicron*, *Bifidobacterium longum* or co-colonized with both demonstrated that these distinct bacterial species impact each other’s transcriptional profile [19]. In some circumstances, bacterial species work together in assembly-line fashion to dismantle complex carbohydrates, where the product of one species becomes the substrate for another [20]. In other circumstances, however, microbial species within the intestine compete for limited nutrients and space in order to persist. For example, similarities in carbon utilization by *Escherichia coli* and *Citrobacter rodentium* lead to competition between these species. Niche specialization also results in competition between the same, but not different, strains of *Bacteroides spp*. [21, 22]. Metabolic overlap and niche restriction, however, can be shared by distinct bacterial species as is the case with *Salmonella typhimurium* and *Clostridium difficile* which benefit from the transient abundance of the same sugars following antibiotic treatment [23]. Yet, the extent to which metabolic competition between antibiotic-resistant microbes contributes to their prevalence and persistence is unknown.

In this study, we investigated the interactions between VRE, *K*. *pneumoniae* and the host in a murine model of intestinal colonization. Our goal was to determine whether intestinal domination by either VRE or *K*. *pneumoniae* would provide colonization resistance against the other. We found that VRE and *K*. *pneumoniae* were able to co-exist despite occupying the same intestinal sites, while restoration of a normal microbiota by means of a fecal transplant displaced both organisms effectively but at different rates. Antibiotic treatment of mice reduced the thickness of the colonic mucin layer, a defect that was corrected by *K*. *pneumoniae* but not VRE colonization. However, *K*. *pneumoniae* was more effective than VRE at invading the mucus layer and translocating to mesenteric lymph nodes. Our findings demonstrate that highly antibiotic-resistant bacteria such as VRE and *K*. *pneumoniae* non-competitively co-occupy the colon but establish distinct relationships with the mucosal epithelium.

## Results

### VRE and *K*. *pneumoniae* coexist in the gastrointestinal tract

**Fig 1:**
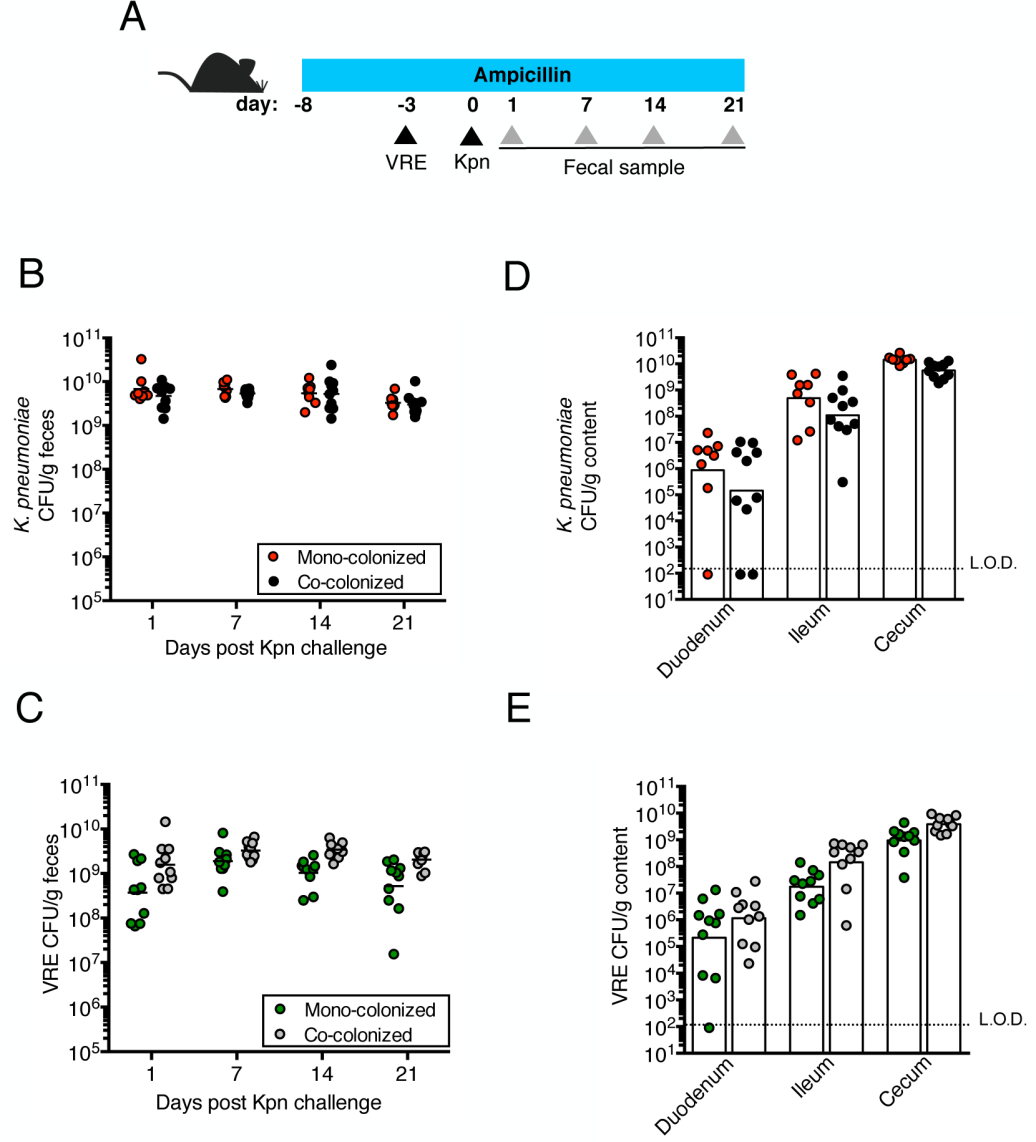
Pre-colonization with VRE does not prevent colonization by *K*. *pneumoniae*. (A) Experimental design. Mice were treated with ampicillin for 29 days. On day 5 of ampicillin treatment, mice were inoculated with 5x10^4^ colony-forming units (CFU) of VRE by oral gavage or left uninfected. Three days later, half of the VRE-infected mice and the uninfected group were challenged with 5x10^4^ CFU of *K*. *pneumoniae* (Kpn). (B, C) CFU of *K*. *pneumoniae* (B) and VRE (C) were quantified in fecal pellets collected at different time points post *K*. *pneumoniae* inoculation. (D, E) Mice were sacrificed 21 days post *K*. *pneumoniae* challenge. *K*. *pneumoniae* (D) and VRE (E) burden was quantified in the luminal contents from the duodenum, ileum and cecum. L.O.D., limit of detection. Data were pooled from two independent experiments (n = 10 per group). (B-E) Data were analyzed by the Mann-Whitney test.

**Fig 2:**
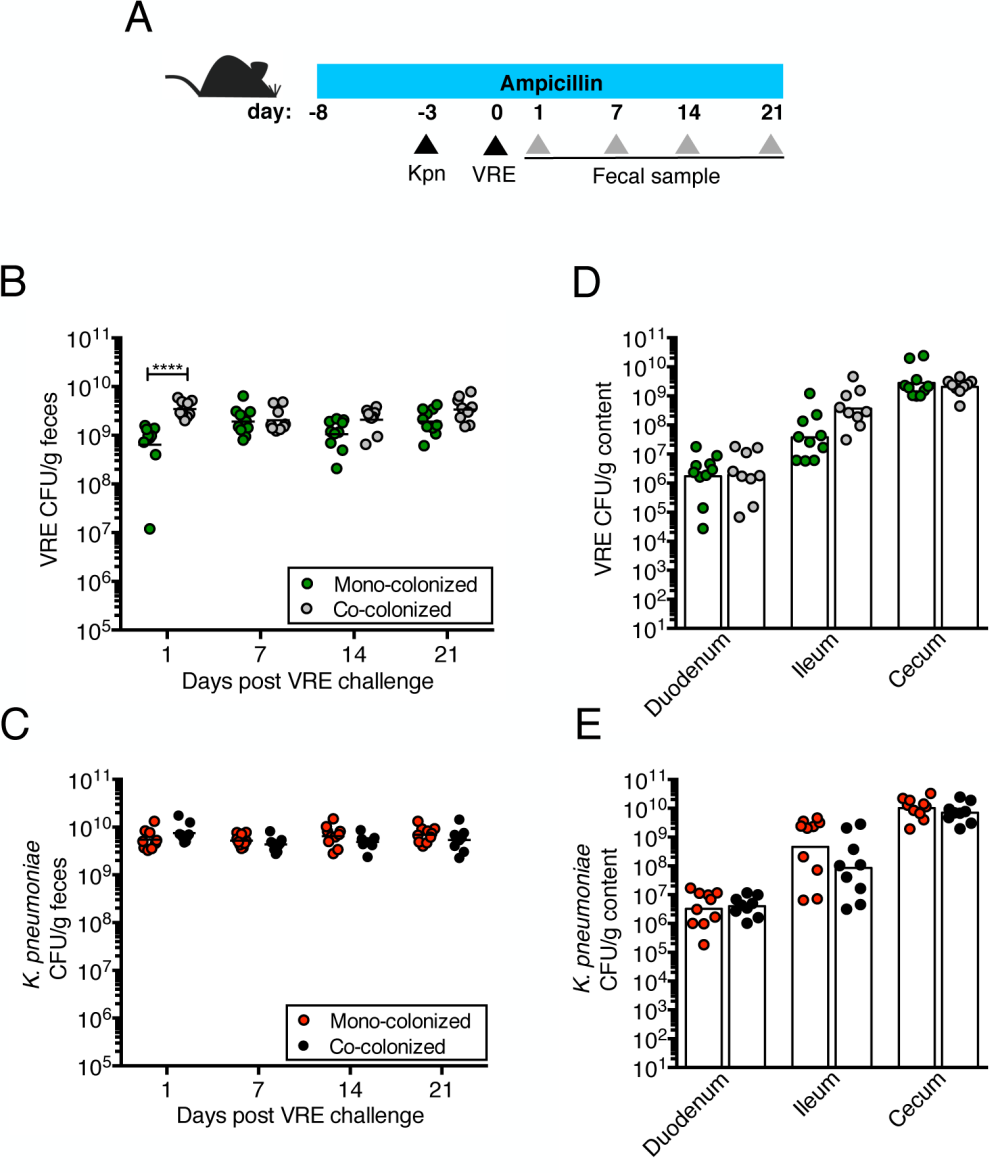
Pre-colonization with *K*. *pneumoniae* does not prevent colonization by VRE. (A) Experimental design. Mice were treated with ampicillin for 29 days. On day 5 of ampicillin treatment, mice were inoculated with 5x10^4^ colony-forming units (CFU) of *K*. *pneumoniae* (Kpn) by oral gavage o left uninfected. Three days later, half of the *K*. *pneumoniae*-infected mice and the uninfected group were challenged with 5x10^4^ CFU of VRE. (B, C) CFU of VRE (B) and *K*. *pneumoniae* (C) were quantified in fecal pellets collected at different time points post VRE inoculation. (D, E) Mice were sacrificed 21 days post VRE challenge. VRE (D) and *K*. *pneumoniae* (E) burden was quantified in the luminal contents from the duodenum, ileum and cecum. L.O.D., limit of detection. Data were pooled from two independent experiments (n = 10 per group). (B-E) ****P<0.0001, by the the Mann-Whitney test.

We previously showed that treatment with ampicillin, a broad-spectrum antibiotic, renders C57BL/6 mice from Jackson Laboratories highly susceptible to VRE colonization [7]. Inoculation of ampicillin-treated mice with *K*. *pneumoniae* also resulted in dense colonization of the colon, with approximately 10^10^ colony-forming units (CFU) per gram of feces (S1B Fig). To assess the impact of VRE and *K*. *pneumoniae* on each other’s ability to colonize the gut, we performed a series of experiments in which ampicillin-treated mice were pre-colonized with VRE or *K*. *pneumoniae* by gastric lavage and challenged with the other species 3 days later, at which point the initial colonizer has become established in the intestine and reached maximum density (S1B Fig). Fecal levels of *K*. *pneumoniae* and VRE were determined by culture beginning one day and continuing through 21 days post infection (p.i.) with the challenge species. We found that dense colonization with VRE did not significantly impact *K*. *pneumoniae* colonization levels at any time point over the course of the experiment. However, fewer *K*. *pneumoniae* were recovered from the feces of co-colonized mice on day 1 p.i., suggesting a lag in establishment of *K*. *pneumoniae* in the presence of VRE shortly after infection (Fig 1B). On day 21, mono- and co-colonized animals had comparable *K*. *pneumoniae* burden in the proximal and distal small intestine (duodenum and ileum, respectively) as well as in the large intestine (cecum) (Fig 1D). Under this experimental condition, introduction of *K*. *pneumoniae* did not reduce VRE density in the intestine. Rather, VRE CFU levels were, for the most part, similar in mono- and co-colonized mice although a trend toward increased VRE burden was observed on days 1, 14 and 21 post challenge (Fig 1C and 1E). In the converse experiment, challenge of *K*. *pneumoniae*-dominated mice with VRE resulted in a transient but significant increase in VRE density 1 day p.i. but then equivalent density in all intestinal compartments when compared to antibiotic-treated mice lacking *K*. *pneumoniae* (Fig 2B and 2D). In addition, we observed no difference in *K*. *pneumoniae* colonization between mice challenged with VRE and mice mono-colonized with *K*. *pneumoniae* (Fig 2C and 2E). Overall, our results demonstrate that VRE and *K*. *pneumoniae* neither compete nor synergize with each other upon dense colonization of the murine gut.

### VRE and *K*. *pneumoniae* achieve similar densities in the large intestine of co-colonized mice

**Fig 3:**
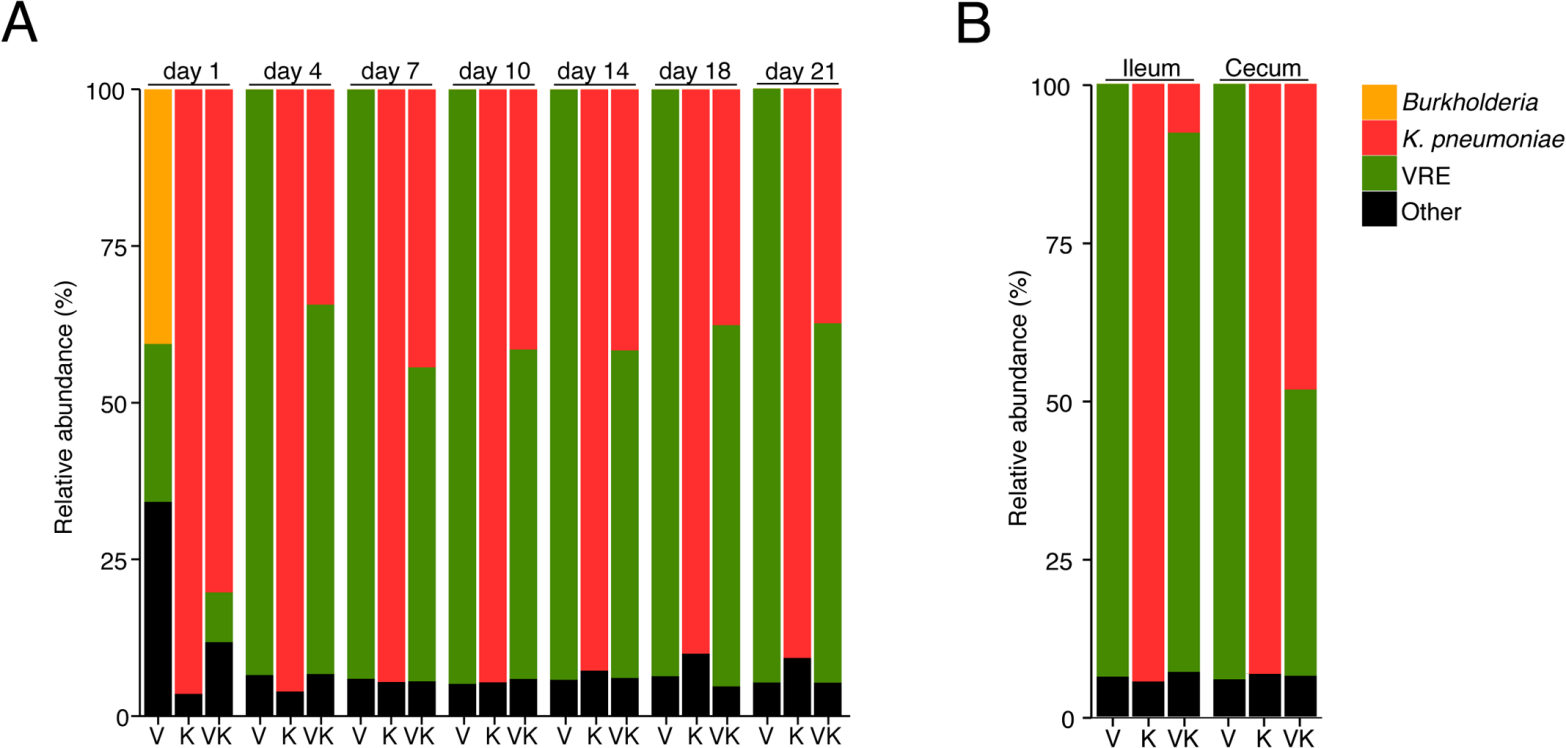
*K*. *pneumoniae* and VRE achieve similar densities in the large intestine of co-colonized mice. Ampicillin-treated mice were inoculated with *K*. *pneumoniae* by oral gavage or left uninfected. Three days later, half of the *K*. *pneumoniae*-infected mice and the uninfected group were challenged with VRE. Microbiota composition of mice colonized with VRE alone (V), *K*. *pneumoniae* alone (K) or both (VK) was determined by sequencing of the V4-V5 region of the 16S rRNA genes. (A) Fecal microbiota composition at different time points post VRE challenge. (B) Ileal and cecal microbiota composition at day 21 of colonization. (A,B) Each stacked bar represents the average of five individually-housed mice per time point.

Because VRE becomes the dominant member of the intestinal microbiota within days after administration to antibiotic-treated mice [7], we examined how colonization by *K*. *pneumoniae* and subsequent VRE challenge would impact their relative proportions at different time points p.i. On day 1 post challenge, VRE represented 30% of the total fecal bacteria in mono-colonized mice, with the remaining 70% constituting bacteria that remained and expanded following antibiotic treatment. In mice previously colonized with *K*. *pneumoniae*, VRE represented 10% of the microbiota and *K*. *pneumoniae* dominated. However, VRE rapidly expanded in the colon of co-colonized animals and within 4 days of inoculation, VRE and *K*. *pneumoniae* achieved roughly equal densities and remained stable for up to 21 days (Fig 3A). This ratio was specific to the large intestine since both bacterial species were equally abundant in the cecum and colon but VRE dominated over *K*. *pneumoniae* in the ileum (Fig 3B).

### Fecal bacteriotherapy eliminates established VRE and *K*. *pneumoniae* intestinal domination

**Fig 4:**
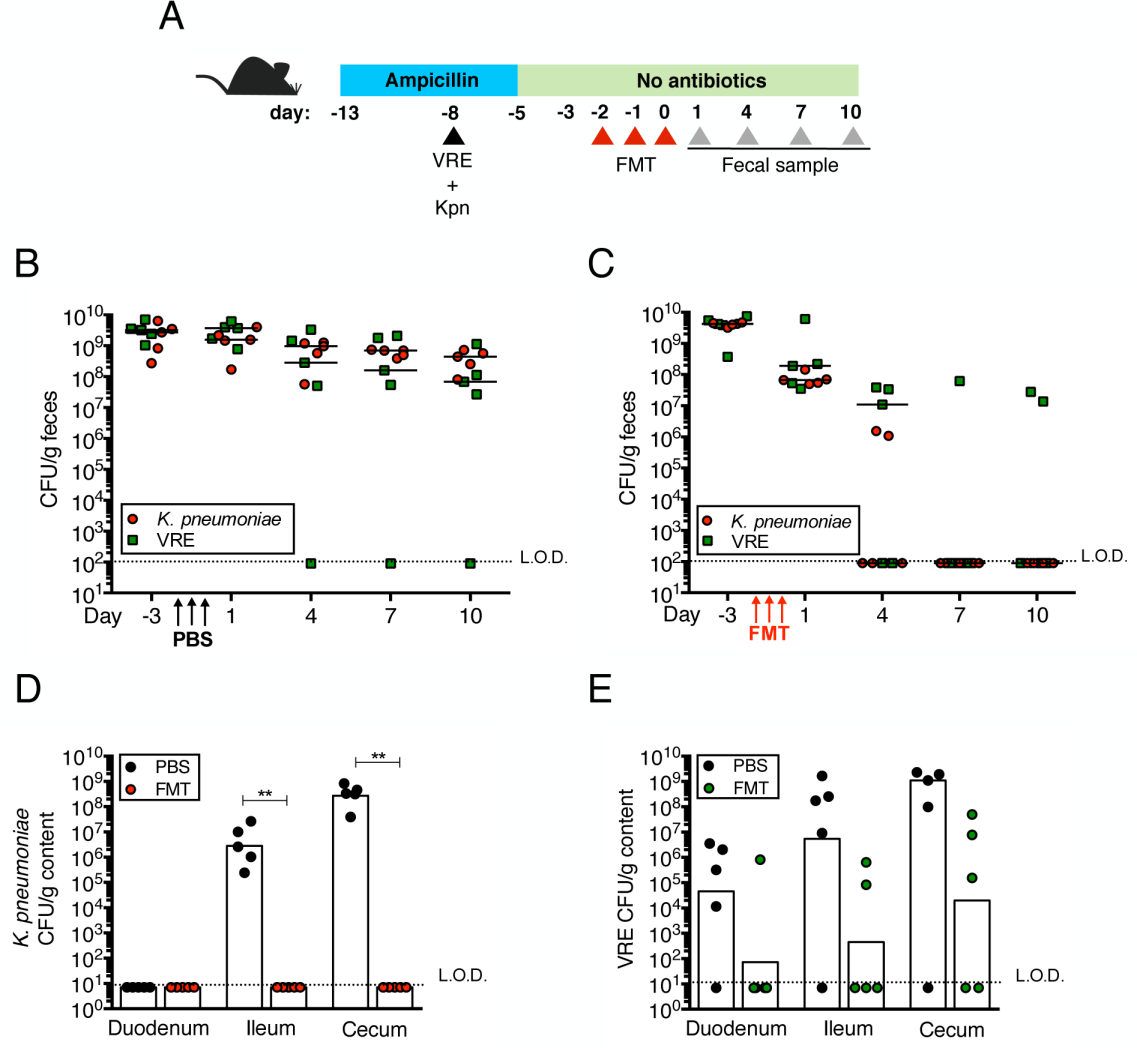
Fecal bacteriotherapy eliminates established *K*. *pneumoniae* and VRE intestinal domination. (A) Experimental design. Mice were treated with ampicillin for 8 days. On day 5 of ampicillin treatment, mice were simultaneously infected with 5x10^4^ CFU of VRE and *K*. *pneumoniae* (Kpn). Three days post infection, ampicillin treatment was stopped. Mice were administered PBS or a fecal microbiota transplant (FMT) from an untreated mouse on three consecutive days starting on the third day after ampicillin cessation. (B, C) VRE and *K*. *pneumoniae* burden was quantified in fecal pellets at the indicated time points after the last PBS (B) or FMT (C) dose. (D, E) PBS- and FMT-treated mice were sacrificed on day 10 following the last treatment dose and numbers of *K*. *pneumoniae* (D) and VRE (E) CFU were quantified in the duodenum, ileum and cecum. L.O.D., limit of detection. n ≥ 5 per group. (B-E) **P<0.005 by the Mann-Whitney test.

Transplantation of feces from donor mice that have not been treated with antibiotics can eliminate VRE from the intestine of densely colonized mice and, in humans, fecal transplantation from healthy donors cures patients with recurrent *Clostridium difficile* infection [11, 24]. To determine whether the kinetics of VRE and *K*. *pneumoniae* clearance from the murine intestine following fecal transplantation are similar or distinct, we colonized ampicillin-treated mice with VRE and *K*. *pneumoniae* concurrently, terminated ampicillin treatment and treated mice with fecal microbiota transplants (FMT) or PBS on three consecutive days (Fig 4A). VRE and *K*. *pneumoniae* colonization levels were similar in the feces before FMT administration and remained elevated in mice that received PBS instead of FMT (Fig 4B). However, following FMT treatment, *K*. *pneumoniae* density in fecal pellets decreased within one day and became undetectable within 7 days in all mice (Fig 4C). VRE, on the other hand, was cleared in 60% of the mice but reduced by 3 logs in the remaining 40%. Increased colonization resistance against *K*. *pneumoniae* as opposed to VRE was also observed in mice that had not been treated with antibiotics (S1A Fig). *K*. *pneumoniae* was also cleared more effectively than VRE from the duodenum, ileum and cecum of FMT-treated animals while the density of these bacterial species remained high in PBS-treated mice (Fig 4D and 4E). These findings suggest that the mechanisms of microbiota-mediated colonization resistance against VRE and *K*. *pneumoniae* are distinct or that *K*. *pneumoniae* is more susceptible to colonization resistance.

### *K*. *pneumoniae* and VRE reside within the same intestinal regions but occupy distinct metabolic niches

**Fig 5:**
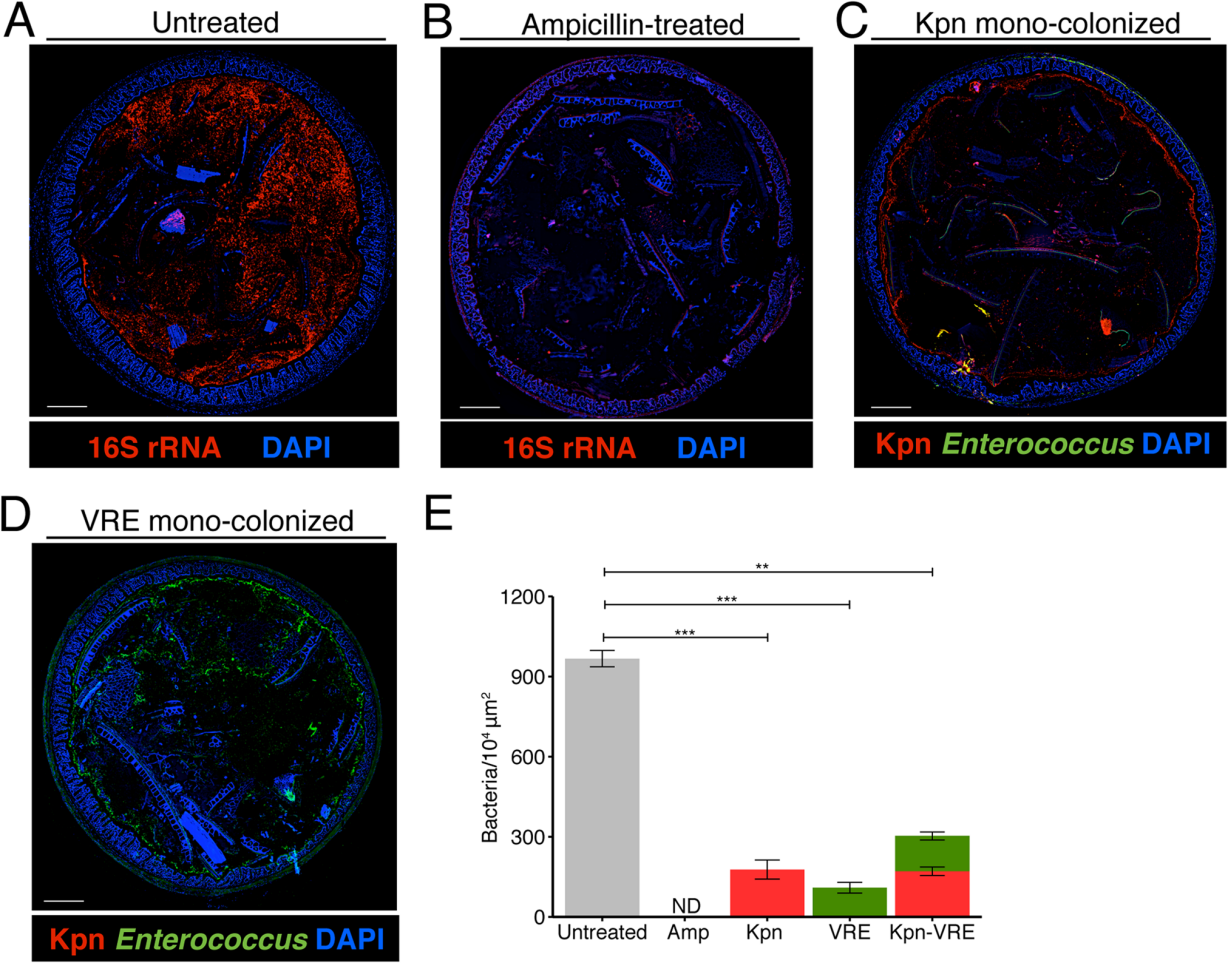
*K*. *pneumoniae* and VRE occupy a fraction of the total available space in the colon. (A-E) Visualization of bacterial localization by FISH. Entire colon cross-sections from untreated mice (A) and mice treated with ampicillin for 3 weeks (B) were stained with a universal probe that targets the 16S rRNA gene of all bacteria. Cross-sections from ampicillin-treated mice colonized with *K*. *pneumoniae* (C) or VRE (D) for 21 days were hybridized with probes specific for *K*. *pneumoniae* (Kpn) and Enterococcus, respectively. Sections were counterstained with Hoechst dye to visualize nuclei. Images are representative of 5 mice per group. Scale bar, 500 μm. (E) Number of bacteria per unit area of whole colon cross-sections. n = 3 per group. ND = non-detectable. Error bars (mean ± SEM). **P<0.005, ***P<0.0005 by the Mann-Whitney test.

**Fig 6:**
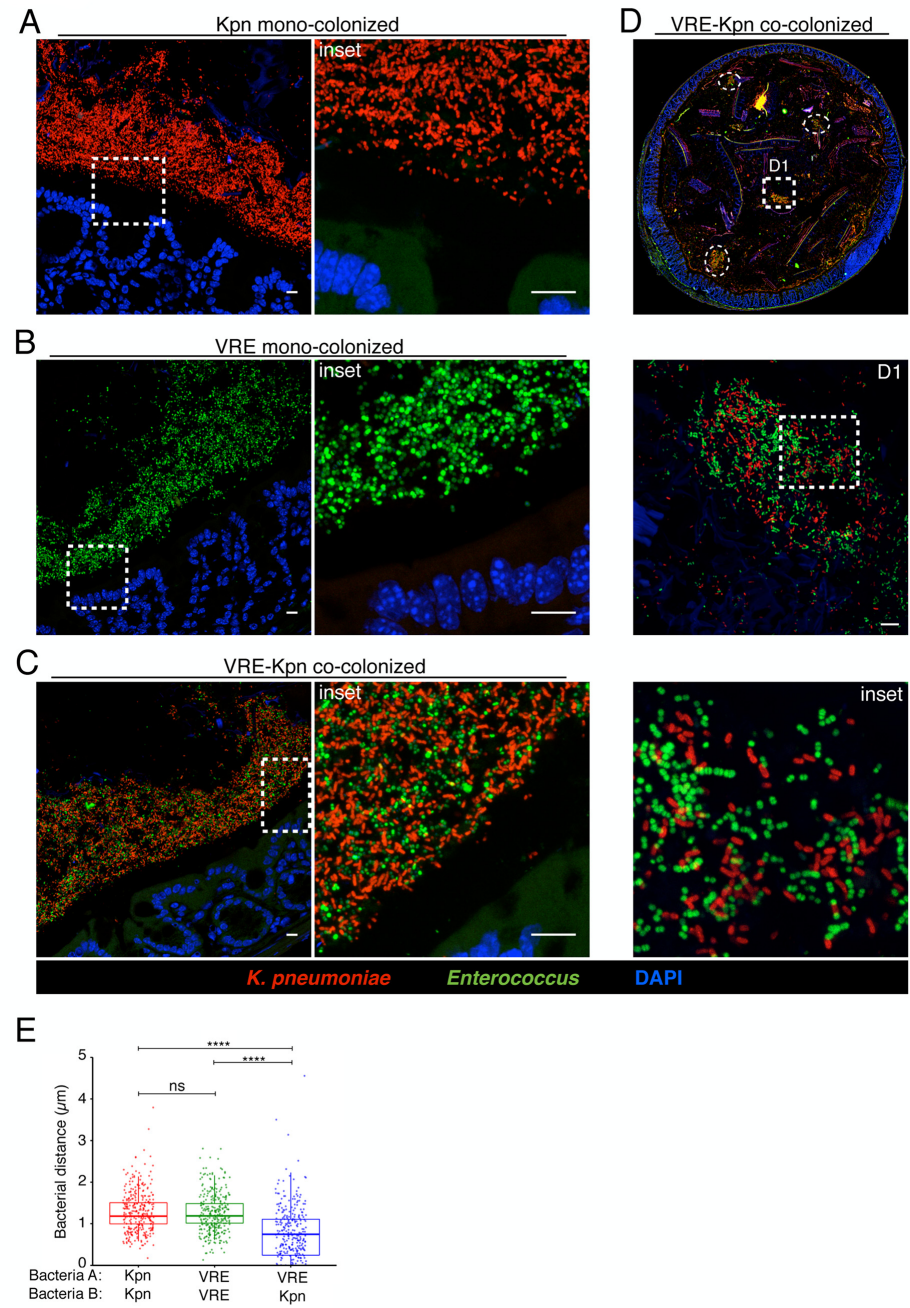
*K*. *pneumoniae* and VRE reside within the same intestinal regions but occupy distinct metabolic niches. (A-D) Spatial localization of *K*. *pneumoniae* and VRE in the colon. Colon sections from ampicillin-treated mice colonized for 21 days with *K*. *pneumoniae* alone (A), VRE alone (B) and *K*. *pneumoniae* together with VRE (C, D) were hybridized with probes specific for *K*. *pneumoniae* and Enterococcus. (D) VRE and *K*. *pneumoniae* islands (dashed circles and square) in the colonic lumen of co-colonized mice. (A-D) All sections were counterstained with Hoechst dye to visualize nuclei. Scale bars, 10 μm. Insets, 63X oil objective plus 4X digital zoom. Images are representative of at least 5 mice per group. (E) Minimum distance between neighboring bacteria determined by confocal microscopy. ns = non-significant; ****P<0.0001, by the Mann-Whitney test.

The findings that *K*. *pneumoniae* and VRE do not interfere with each other’s ability to colonize the gut lumen and that their elimination from the intestine following FMT differs suggest that these bacterial species occupy distinct intestinal niches. To localize bacteria within the colons of mice we performed fluorescence *in situ* hybridization (FISH) with a universal probe targeting bacterial 16S rRNA genes. In mice that had not been treated with antibiotics, we detected a dense and morphologically diverse bacterial microbiota that was almost completely depleted by ampicillin-treatment (Figs 5A, 5B, S2A and S2B). FISH analysis of antibiotic-treated mice colonized with *K*. *pneumoniae* (Kpn), VRE or both with species-specific oligonucleotide probes revealed that *K*. *pneumoniae* and VRE were most abundant in luminal areas adjacent to the colonic epithelial layer and that both organisms localized to the same intestinal sites (Figs 5C, 5D and 6A–6D).

Confocal microscopy-based quantification of VRE and *K*. *pneumoniae* in mono-colonized mice demonstrated that *K*. *pneumoniae* and VRE each only achieved 10% of the bacterial density detected in antibiotic-naive mice with a diverse microbiota (Fig 5E). In co-colonized mice, the densities of VRE and *K*. *pneumoniae* in the colonic lumen were additive, supporting the idea that VRE and *K*. *pneumoniae* do not interfere with one another and that their metabolic needs may differ (Fig 5E). Furthermore, *K*. *pneumoniae* and VRE were generally occupying overlapping regions within luminal areas closest to the colonic epithelium, although islands of increased bacterial density were also detected more centrally in the colonic lumen (Fig 6C and 6D). To further assess whether VRE and *K*. *pneumoniae* compete for space within the regions they occupy in the colon, we measured distances between neighboring VRE bacteria, between neighboring *K*. *pneumoniae* bacteria and between neighboring VRE and *K*. *pneumoniae* bacteria. Supporting the notion that VRE and *K*. *pneumoniae* occupy different metabolic niches, intraspecies distances were significantly greater than interspecies distances between VRE and *K*. *pneumoniae*, suggesting that localized nutrient depletion may promote spatial avoidance among competing bacteria while non-competing bacteria ignore each other.

### Antibiotic treatment and colonization with *K*. *pneumoniae* or VRE influences the thickness and integrity of the inner mucus layer

**Fig 7:**
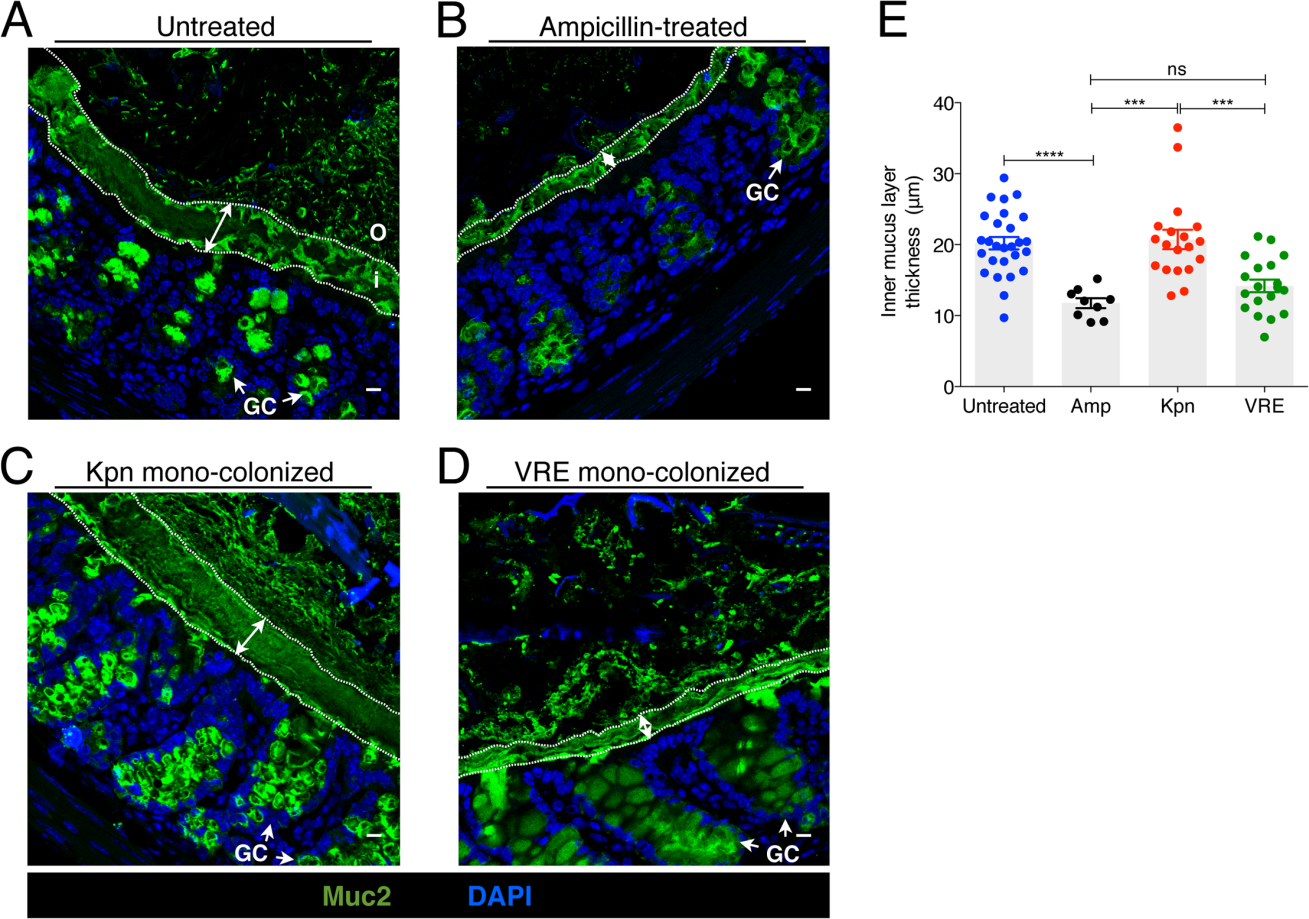
*K*. *pneumoniae* and VRE colonization influences the thickness of the inner mucus layer. (A-D) Colon sections from untreated mice (A), ampicillin-treated mice (B) and ampicillin-treated mice mono-colonized with either *K*. *pneumoniae* (C) or VRE (D) for 21 days were stained with an anti-Muc2 antibody to visualize the inner and outer mucus layers along with goblet cells (arrows). Double arrows denote the inner mucus layer. i, inner mucus layer; o, outer mucus layer; GC, goblet cell. Scale bars, 10 μm. Images are representative of 5 mice per group. (E) Quantification of inner mucus layer thickness. Error bars (mean ± SEM). n = 3–6 mice per group. ns = non-significant; ***P<0.0005, ****P<0.0001, by the unpaired Student *t* test.

The murine colonic mucus layer consists almost exclusively of the mucin Muc2 [25]. Upon release from goblet cells, Muc2 forms a gel-like layer that coats the intestinal epithelium and that is largely impenetrable to bacteria. Beyond the thick, epithelium-associated mucin layer is a loosely attached and less dense mucin layer that is readily penetrated by intestinal bacteria and that serves as a microbial habitat [25, 26]. Under normal homeostatic conditions, bacterial molecules released by the microbiota and host factors regulate the production and secretion of mucus [27] and thinning of the colonic mucus layer occurs following antibiotic treatment [18]. The infectivity of some intestinal pathogens, such as *C*. *rodentium* and *Salmonella enterica* sv Typhimurium, is enhanced by disruption of the mucus layers in the colon [15, 28]. To explore the impact of *K*. *pneumoniae* and VRE colonization on the colonic mucus layer, we stained colonic sections for Muc2 and measured the thickness of the inner mucus layer. While the dense mucus layer was significantly reduced in mice treated with ampicillin (Fig 7A, 7B and 7E), mono-colonization of ampicillin-treated mice with *K*. *pneumoniae* but not VRE resulted in recovery of normal mucus layer thickness (Fig 7C–7E). This indicates that bacterial species differ in their ability to promote mucin production and mucus layer generation in the murine colon.

### *K*. *pneumoniae* and VRE differ in their ability to invade the colonic mucus barrier and translocate to extra-intestinal sites

**Fig 8:**
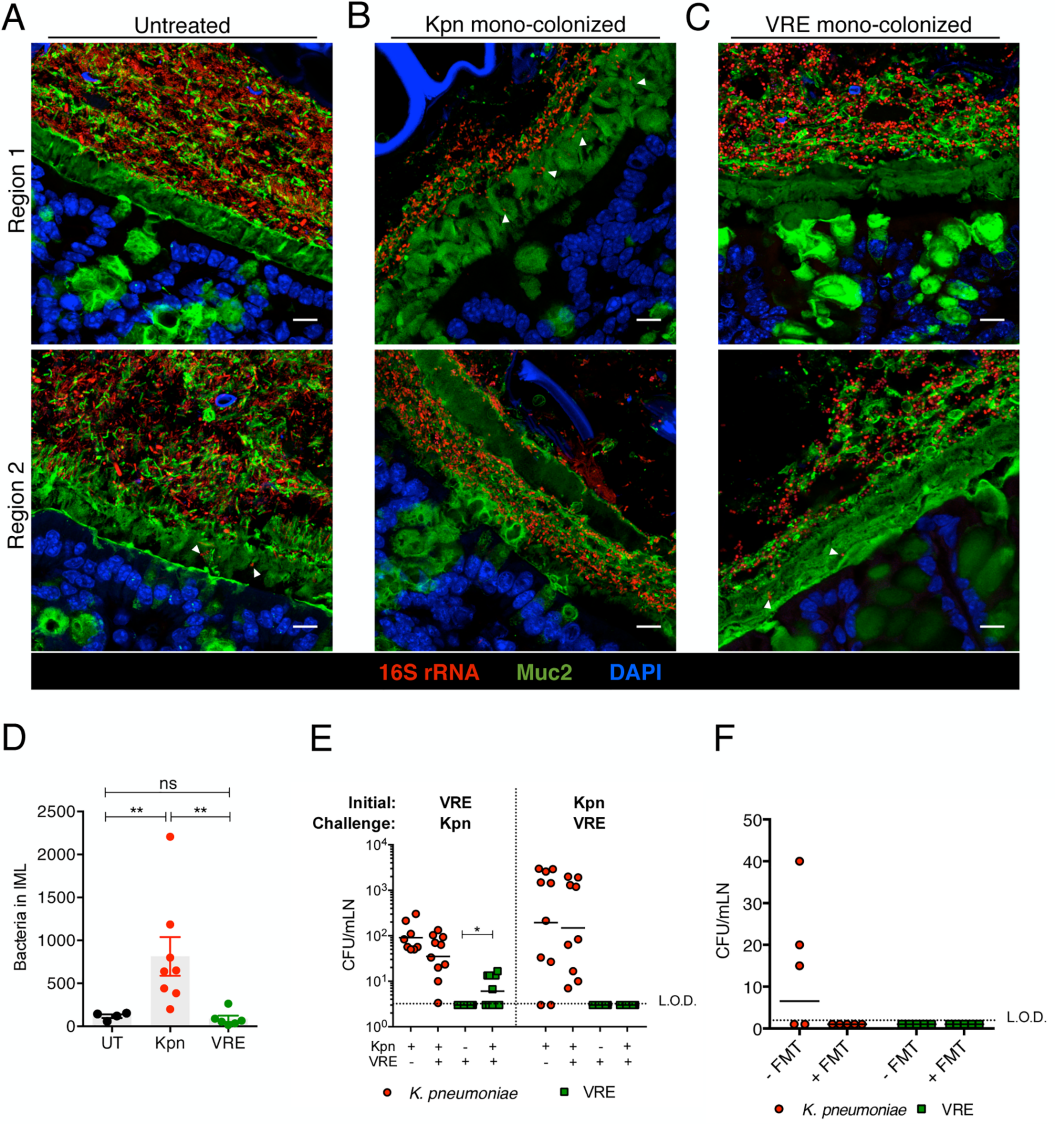
Differential mucus layer infiltration and translocation by VRE and K. *pneumoniae*. (A-C) Dual immunostaining of colon sections from untreated mice (A) and ampicillin-treated mice mono-colonized with either *K*. *pneumoniae* (B) or VRE (C) for 21 days using anti-Muc2 and a pan-bacterial 16S rRNA gene FISH probe. Sections were counterstained with Hoechst dye to visualize nuclei. Arrowheads indicate bacteria within the inner mucus layer. Scale bar, 10 μm. Images are representative of 5 mice per group. Boundaries of the inner mucus layer (IML) zone were determined by the density of Muc2 staining and the stratified organization characteristic of the inner, but not outer, mucus layer. (D) Number of bacteria within the IML. UT, untreated. (E) Numbers of VRE and *K*. *pneumoniae* in mesenteric lymph nodes of mono-colonized mice and mice pre-colonized with either VRE or *K*. *pneumoniae* (initial strain) and challenged with the opposite strain at day 21 post challenge. Data were pooled from two independent experiments. (F) Numbers of VRE and *K*. *pneumoniae* in mesenteric lymph nodes of mice co-colonized with VRE and *K*. *pneumoniae* with or without a fecal transplant (FMT) 10 days after receiving the last of three FMT/PBS doses. Data were pooled from two independent experiments. L.O.D., limit of detection. (D-F) ns = non-significant; *P<0.05, **P<0.005, by the Mann-Whitney test.

To investigate the interactions between *K*. *pneumoniae* and VRE with the colonic mucin layer, we mono-colonized ampicillin-treated mice and performed FISH in conjunction with Muc2 immunostaining to localize bacteria in relation to the inner mucus layer (IML). Very few bacteria were detected within the IML in antibiotic-naïve mice harboring a diverse microbiota (Fig 8A). On the other hand, *K*. *pneumoniae* was detected within the IML in mono-colonized mice, with the extent of mucin penetration varying in different regions of a colonic cross-section (Fig 8B). VRE also penetrated the IML at different regions but to a lesser extent than *K*. *pneumoniae* (Fig 8C). Image analysis of entire colon cross-sections revealed substantially higher bacterial numbers in the IML of *K*. *pneumoniae* mono-colonized mice compared to antibiotic-naïve or VRE mono-colonized animals (Fig 8D). Within the colonic lumen, however, *K*. *pneumoniae* and VRE were similarly associated with detached islands of mucus (Fig 8B and 8C).

To determine whether increased mucus layer penetration by *K*. *pneumoniae* is associated with increased invasion of deeper tissues, we cultured mesenteric lymph nodes (mLNs). In mono-colonized mice, we detected up to 10^4^ live *K*. *pneumoniae* in mLNs three weeks p.i. (Fig 8E) Notably, infection of mLNs by *K*. *pneumoniae* was not affected by co-colonization with VRE, regardless of the order of microbial administration. In contrast, we did not recover any live bacteria from mLNs of VRE mono-colonized animals, perhaps reflecting their reduced penetration of the IML. However, challenge of VRE mono-colonized mice with *K*. *pneumoniae* resulted in VRE translocation to mLNs in 60% of the mice, suggesting that introduction of *K*. *pneumoniae* may enhance the ability of VRE to traverse the mucin layer and gain access to the intestinal epithelium (Fig 8E). Administration of fecal microbiota transplants to colonized mice reduced *K*. *pneumoniae* infection of mLNs (Fig 8F), suggesting that isolation of live bacteria from mLNs of *K*. *pneumonia*e-colonized mice results from continuous seeding of the node rather than bacterial growth and persistence in mLNs. Our findings demonstrate that *K*. *pneumoniae*, upon achieving high density in the intestine, can penetrate the dense mucin layer of the colon. In contrast, VRE access to the epithelial layer appears to be more restricted by the mucin layer. Although the ability to penetrate mucus has been associated with pathogenic bacteria, our results suggest that among commensal bacterial species mucus penetration may correlate with their ability to disseminate beyond the gut lumen.

## Discussion

VRE and *K*. *pneumoniae* are two of the most common highly antibiotic- resistant bacterial species that cause infections in hospitalized patients. Both organisms are oxygen-tolerant facultative anaerobes and principally reside in the lower gastrointestinal tract where, under normal circumstances, they are minor contributors to a colonic microbiota composed predominantly of oxygen-intolerant obligate anaerobes. Microbiota analyses have revealed that some patients undergoing allogeneic hematopoietic stem cell transplantation have marked expansion of VRE or *K*. *pneumoniae* in their colonic microbiota [7, 8], to the point where in many cases these species constitute the overwhelming majority of the intestinal bacterial taxa. Our experiments with murine models demonstrate that both organisms can undergo massive expansion in the gastrointestinal tract of antibiotic-treated mice, occupy the same intestinal regions and coexist without exerting any colonization resistance against each other.

The mechanisms that determine bacterial density in the colon remain incompletely defined. During broad-spectrum antibiotic treatment the density of bacteria in the colon, as determined by quantitative 16S rRNA gene PCR, is reduced over 1,000 fold. Nevertheless, upon termination of antibiotic treatment, the compositionally-restricted residual flora re-expands to a density that is similar to pre-treatment levels [7, 29]. Along similar lines, we find that VRE and *K*. *pneumoniae* achieve densities of 10^10^ CFU per gram of colonic content in antibiotic-treated mice, at which point their growth stops, potentially resulting from nutrient depletion or quorum sensing mechanisms that remain undefined [30]. Because VRE and *K*. *pneumoniae* grow independently in the colons of antibiotic-treated co-colonized mice, our findings suggest that distinct mechanisms determine maximal bacterial density of these two bacterial species.

Competition for resources has been demonstrated in several *in vivo* colonization studies involving bacteria of the same or closely related species [21, 22, 31, 32]. In the intestine, heavily glycosylated mucus is a major source of energy for bacteria. Commensals that express mucus-degrading enzymes, such as *Bacteroides thetaiotaomicron*, can cleave sugars from host glycans and support the growth of other members of the colonic microbiota [23, 33]. Enterococci and γ-Proteobacteria do not express glycosidases that degrade mucosal polysaccharides, and thus their carbohydrate utilization is limited to less complex sugars [34, 35]. Some studies have demonstrated that mucus-derived carbohydrates, including sialic acid and fucose, transiently accumulate following antibiotic-treatment, presumably as a result of hydrolases released during bacterial cell lysis, and promote the growth of *Salmonella* and *Clostridium difficile* [23, 36]. However, given the transient release of these carbohydrates following initiation of antibiotic treatment, it is unlikely that this process is sustaining dense and prolonged colonization by either VRE or *K*. *pneumoniae*. Although *E*. *faecium* and *K*. *pneumoniae* can metabolize monosaccharides, and the range of potential carbon sources for *K*. *pneumoniae* is broad [37], the *in vivo* carbohydrate dependencies of these two bacterial species remain largely uncharacterized. The lack of competition between VRE and *K*. *pneumoniae* could potentially be explained by a difference in metabolic requirements (i.e. different sugar utilization) and the observation that bacteria can switch to alternative nutrient sources in the presence of competing strains [38]. Further studies that examine the metabolic profiles of VRE and *K*. *pneumoniae* during mono- and co-colonization will be necessary to identify the mechanisms determining their density in the intestinal tract.

In addition to serving as a nutrient source for intestinal bacteria, mucus also provides a barrier that keeps bacteria away from the colonic epithelium. The intestinal microbiota induces mucin production and antibiotic administration results in thinning of the mucus barrier, thereby increasing susceptibility to bacterial invasion [18]. Pathogens such as *Salmonella* and *C*. *rodentium* can penetrate the mucus layer and in the process induce increased mucin production, a host defense mechanism that limits bacterial invasion [15, 28]. Consistent with these studies, we find that *K*. *pneumoniae* infiltrated the mucus layer while also restoring its thickness to pre-antibiotic treatment levels. VRE colonization of antibiotic-treated mice, on the other hand, did not induce mucus layer thickening. In addition, VRE did not invade the mucus layer to the same degree as *K*. *pneumoniae*. It remains to be determined whether enhanced mucus production is a consequence of bacterial invasion or rather, molecular differences between Gram-negative and Gram-positive bacteria.

In mice with a normal flora and intact TLR signaling, the dense mucus layer is largely devoid of bacteria [17, 25]. Antibiotic treatment, however, reduces the expression of antimicrobial molecules such as RegIIIγ [14], potentially enabling bacteria to gain access to and survive within the mucus layer. Therefore, it is possible that a paucity of host-derived antimicrobial proteins in the mucin layer renders it more penetrable. A recent study showed that mice harboring high levels of bacteria belonging to the Proteobacteria and TM7 phyla have an inner mucus layer that is normal in thickness but penetrable to bacteria [39]. Thus, it is also possible that *K*. *pneumoniae* may induce mucins with abnormal glycosylation or that are structurally disorganized.

Although intestinal colonization with VRE and *K*. *pneumoniae* is asymptomatic, it increases the risk of extra-intestinal infection, including bacteremia [8, 40]. Consistent with the observed differences in mucus infiltration, we found increased *K*. *pneumoniae* translocation to mesenteric lymph nodes (mLNs) relative to VRE. Co-colonization with *K*. *pneumoniae* resulted in increased VRE translocation to mLNs, suggesting that *K*. *pneumoniae* may have opened barriers for the less invasive VRE. The high levels of *K*. *pneumoniae* observed in mLNs of intestinally dominated mice and the complete reduction of bacteria following FMT-mediated intestinal clearance of *K*. *pneumoniae* suggests that bacteria are not replicating in the mLNs but rather are delivered on a continuous basis. Although it is unclear how live bacteria are delivered to draining mLNs, recent studies with other bacterial pathogens suggest that cells within the colonic lamina propria, including CD103^+^ and CX3CR1^+^ dendritic cells, may capture *K*. *pneumoniae* from the luminal environment and carry them to mLNs [41, 42].

Enterococci and γ-Proteobacteria constitute a minor population within the human and murine microbiota and are, for the most part, harmless to the host unless intestinal homeostasis is perturbed [26]. In our murine model, intestinal colonization with VRE and *K*. *pneumoniae* did not result in detectable inflammation of the intestinal wall. In clinical scenarios, however, dense intestinal colonization with antibiotic-resistant bacteria is an important risk factor for systemic infection and for patient-to-patient spread. We found that introduction of a normal microbiota into VRE and *K*. *pneumoniae* co-colonized mice resulted in reduction and clearance of both bacterial species, albeit at different rates. Current studies are focusing on the identification of commensal bacterial species that mediate clearance of antibiotic-resistant bacteria. Overall, the findings presented here uncovered previously unrecognized features of VRE and *K*. *pneumoniae* colonization and provide insight into the nature of pathogen coexistence, dissemination and ways to eradicate colonization.

## Materials and Methods

### Mice, bacterial strains and infection

All experiments were carried out using 6–8 week-old C57BL/6 female mice purchased from Jackson Laboratories and housed in sterile cages with irradiated food and acidified water. For experiments involving antibiotic treatment, 0.5g/L ampicillin (Fisher) was administered to animals in the drinking water and changed every 4 days. Vancomycin-resistant *Enterococcus faecium* was purchased from ATCC (700221). Carbapenem-resistant *Klebsiella pneumoniae* was obtained from the Clinical Microbiology Laboratory at Memorial Sloan-Kettering Cancer Center and isolated from blood cultures of a patient. Both bacterial strains were grown at 37°C in Brain Heart Infusion (VRE) or Luria-Bertani (*K*. *pneumoniae*) broth to early stationary phase and diluted in phosphate buffered solution (PBS) to 10^5^ colony-forming units (CFU). For infection experiments, 5x10^4^ CFU of VRE and/or *K*. *pneumoniae* were administered by oral gavage in a 200μl volume on the fifth day of ampicillin treatment. For simultaneous infection of VRE and *K*. *pneumoniae* (Fig 4), inocula were mixed in a 1:1 ratio prior to administration. Mice were single-housed at the time of infection and kept on ampicillin until the end of the experiment unless otherwise specified. Animals were maintained in a specific pathogen-free facility at Memorial Sloan-Kettering Cancer Center. All mouse handling, cage changes and tissue collection were performed in a biosafety level 2 facility wearing sterile gowns, masks and gloves.

### Quantification of VRE and *K*. *pneumoniae* burden

Fecal samples and intestinal contents from the duodenum, ileum and cecum were weighed and resuspended in 1 ml of PBS. Tenfold dilutions were plated on Difco Enterococcosel (supplemented with 8 μg/ml vancomycin; Novaplus and 100 μg/ml streptomycin; Fisher) and Luria-Bertani (supplemented with 50 μg/ml neomycin; Sigma-Aldrich and 100 μg/ml carbenicillin; LabScientific) agar plates for the specific detection of VRE and *K*. *pneumoniae*, respectively. Mesenteric lymph nodes were mashed through a 40 μm filter, resuspended in PBS and plated directly onto selective plates.

### Fecal microbiota transplantation (FMT)

A fecal pellet from an untreated C57BL/6 mouse was resuspended in 1ml of PBS under anaerobic conditions and 200μl of the fecal suspension was administered per mouse via oral gavage starting on the third day of ampicillin cessation. This process was repeated on the fourth and fifth days for a total of three consecutive FMT doses.

### DNA extraction, V4-V5 16S rRNA gene amplification, multiparallel sequencing and sequence analysis

Fecal samples and intestinal contents were frozen immediately after collection in a dry ice/ethanol slurry and stored at -80°C. DNA was extracted using the phenol-chloroform and bead beating method described previously [7]. The V4-V5 region of the 16S rRNA gene was amplified and sequenced with the Illumina Miseq platform as described previously [43]. Sequences were analyzed using version 1.33.3 of the MOTHUR pipeline [44] as described in Buffie et al., 2015. Sequences with distance-based similarity of at least 97% were assigned the same OTU (operational taxonomic unit) and representative OTUs were classified using a modified Greengenes reference database [45].

### Tissue preparation for histology analysis

Intestinal tissues with luminal contents were carefully excised and fixed in freshly made nonaqueous Methacarn solution (60% methanol, 30% chloroform and 10% glacial acetic acid) as previously described [17, 46] for 6 hours at 4°C. Tissues were washed in 70% ethanol, processed with Leica ASP6025 processor (Leica Microsystems) and paraffin-embedded by standard techniques. 5-μm sections were baked at 56°C for 1 hour prior to staining.

### Fluorescence *in-situ* hybridization (FISH)

The hybridization method was adapted from Swidsinski et al., 2005 and Vaishnava at el., 2011. Briefly, tissue sections were deparaffinized with xylene (twice, 10 min each) and rehydrated through an ethanol gradient (95%, 10 min; 90%, 10 min) to water. Sections were incubated with a universal bacterial probe directed against the 16S rRNA gene or with probes specific to *K*. *pneumoniae* and Enterococcus at 50°C for 3 hours. Probes were diluted to 5ng/μl in 0.9M NaCl, 20mM Tris-HCl at pH7.2 and 0.1% SDS prior to use. Sections were later washed twice in 0.9M NaCl, 20mM Tris-HCl at pH7.2 (wash buffer) for 10 min and counterstained with Hoechst (1:3000 in wash buffer) for nuclear staining. The following FISH probes were used: universal bacterial probe EUB338: [Cy3]-GCTGCCTCCCGTAGGAGT-[AmC7~Q+Cy3es] [47]. *K*. *pneumoniae*-specific probe Kpn: [Cy3]-CCTACACACCAGCGTGCC- [AmC7~Q+Cy3es] (probe base accession number 00352, [48]); Enterococcus-specific probe Enfm93: [AminoC6+Alexa488]- GCCACTCCTCTTTTTCCGG-[AmC7~Q+Alexa488] [49].

### Muc2 immunofluorescence

Muc2 immunostaining was performed as previously described [25, 50] with some modifications. Briefly, deparaffinized sections were incubated in 0.9M NaCl, 20mM Tris-HCl at pH7.2 and 0.1% SDS at 50°C for 3 hours, rinsed in PBS and blocked with 5% goat serum in PBS for 30 min at room temperature to minimize non-specific binding. Sections were then washed in PBS for 10 min prior to overnight incubation at 4°C with an anti-Muc2 rabbit polyclonal antibody (H300, Santa Cruz; 1:200 in PBS) [51]. Following incubation with primary antibody, tissues were washed 3 times in PBS for 10 min and incubated with goat-anti-rabbit Alexa 488 secondary antibody (Life Technologies, 1:1000 in PBS) for 1 hour at room temperature. Sections were washed twice in PBS for 10 min and counterstained with Hoechst (1:3000 in PBS). For FISH-Muc2 dual staining, sections were briefly rinsed in wash buffer after FISH hybridization and incubated directly with the anti-Muc2 primary antibody diluted in wash buffer. Incubation with secondary antibody was carried out at 4°C for 2 hours. A single 10 min PBS wash was performed after incubation with the primary and secondary antibodies before Hoechst nuclear staining and mounting with Mowiol solution.

### Microscopy

Images were acquired with a Leica TCS SP5-II upright confocal microscope using a 63x oil immersion lens (NA 1.4, HCX PL APO) with or without digital zoom as a series of short Z-stacks. Maximum intensity projection processing of Z-stacks was done in Fiji (ImageJ) software. Mucus layer thickness was measured using the Leica distance measurement tool (LASAF). The width of the inner mucus layer was determined by the average of 4 measurements per field with 4 fields measured per section. Whole tissue images were digitally scanned using the Zeiss Mirax Desk Scanner with 20x/0.8NA objective. Bacterial distance analysis was performed on colon images taken at 63x magnification with a 4x digital zoom by determining the XY coordinates of each bacterial cell in MetaMorph (Molecular Devices) software and measuring the distance from their center. For quantification of bacterial density and invasion into the mucus layer, whole tissue cross-sections were tile scanned in short Z-stacks using an inverted laser scanning confocal Zeiss LSM 5-Live microscope at 63x magnification. For bacterial quantification, a threshold based on the RGB color combination and intensity of each bacterial species was generated with the color thresholding option in MetaMorph. Thresholded objects of 1μm in size were counted as a single bacterial cell with MetaMorph’s integrated morphometric analysis tool.

### Statistics

The Mann-Whitney test was used for statistical analysis of intestinal CFU as well as image-based quantification of bacterial distance, density and infiltration into the mucus layer. Differences in mucus layer thickness were analyzed with the unpaired Student *t* test. All statistical tests were performed using the Graph-Pad Prism software package (version 6.0). *P* values less than 0.05 were considered to be significant.

### Ethics statement

All mouse experiments were performed in accordance with and approved by the Memorial Sloan-Kettering Institutional Animal Care and Use Committee (IACUC) under protocol 00–05–066. The Memorial Sloan-Kettering IACUC adheres to provisions of the Animal Welfare Act.

## Supporting Information

S1 Fig Ampicillin treatment leads to VRE and *K*. *pneumoniae* expansion in the intestine. Untreated mice (A) and mice treated with ampicillin in the drinking water for 1 week (B) were colonized with 10^8^ CFU of VRE or *K*. *pneumoniae* and bacterial burden was quantified in the feces on days 1, 3 and 7 post infection. Ampicillin-treated animals were kept on ampicillin for the duration of the experiment. L.O.D., limit of detection.
(TIF)

S2 Fig Visualization of morphologically distinct intestinal bacteria by FISH. Colon sections from untreated mice (A) and mice treated with ampicillin in the drinking water for 3 weeks (B) were hybridized with a universal bacterial probe directed against the 16S rRNA gene and counterstained with Hoechst dye to visualize nuclei. Scale bars, 10 μm. Inset, 63X oil objective plus 4X digital zoom. Images are representative of 5 mice per group.
(TIF)
