# Supplementary figures and images for: Distinct but Spatially Overlapping Intestinal Niches for Vancomycin-Resistant Enterococcus faecium and Carbapenem-Resistant Klebsiella pneumoniae
Source: PLoS Pathog. 2015 Sep 3;11(9):e1005132. doi: 10.1371/journal.ppat.1005132 (PMC4559429; doi:10.1371/journal.ppat.1005132)

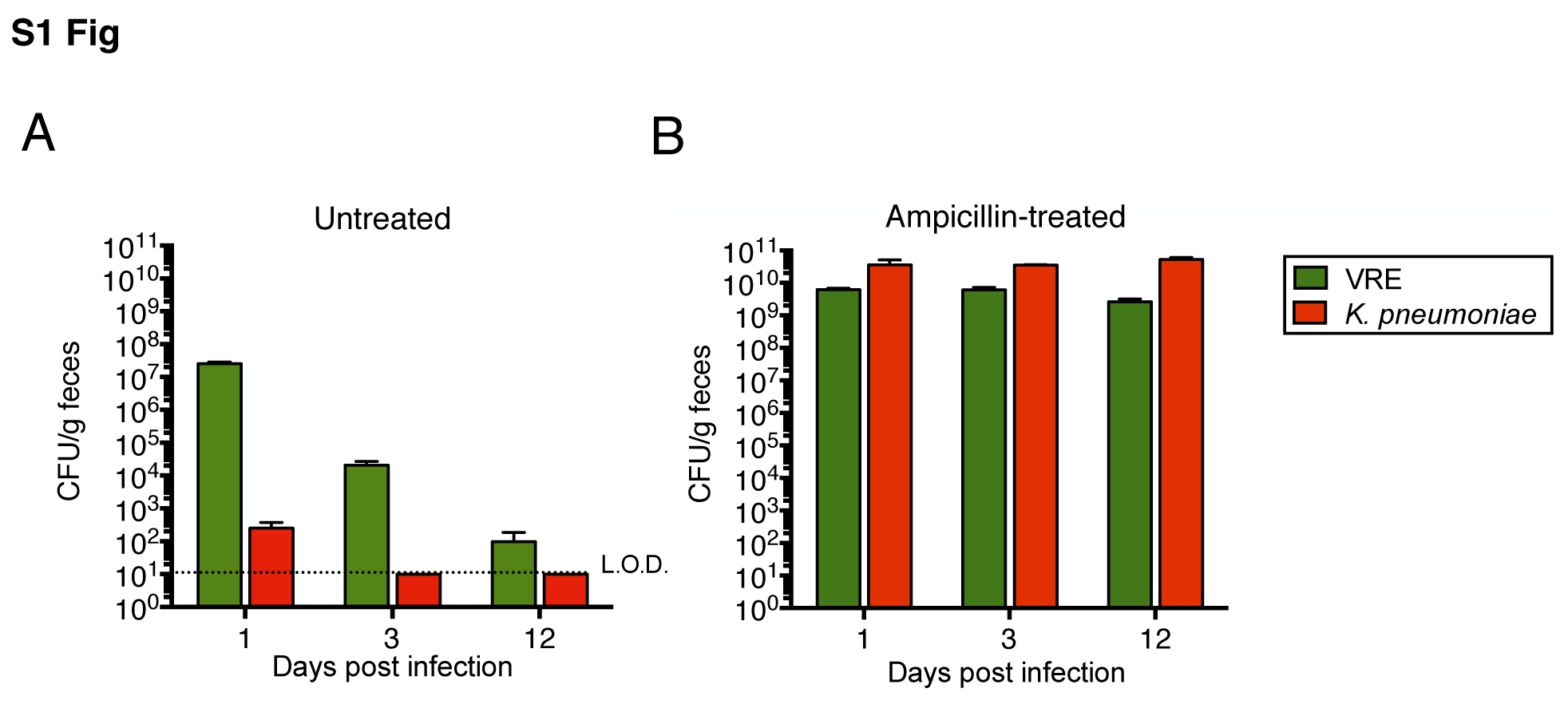

Supplement: S1 Fig — Untreated mice (A) and mice treated with ampicillin in the drinking water for 1 week (B) were colonized with 108 CFU of VRE or K. pneumoniae and bacterial burden was quantified in the feces on days 1, 3 and 7 post infection. Ampicillin-treated animals were kept on ampicillin for the duration of the experiment. L.O.D., limit of detection. (TIF) [file ppat.1005132.s001.tif]

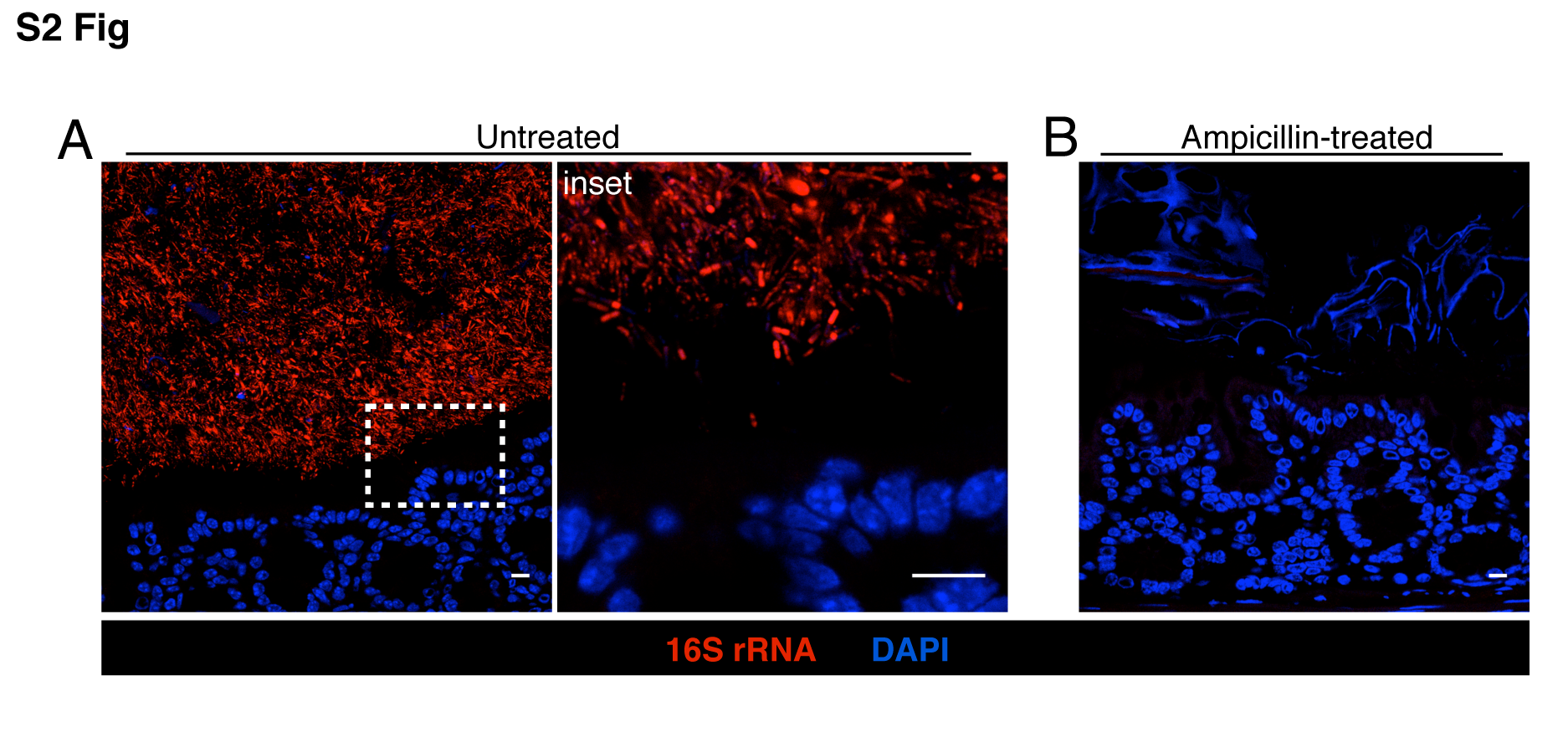

Supplement: S2 Fig — Colon sections from untreated mice (A) and mice treated with ampicillin in the drinking water for 3 weeks (B) were hybridized with a universal bacterial probe directed against the 16S rRNA gene and counterstained with Hoechst dye to visualize nuclei. Scale bars, 10 μm. Inset, 63X oil objective plus 4X digital zoom. Images are representative of 5 mice per group. (TIF) [file ppat.1005132.s002.tif]
